# Characterization of Particle Size Effects on Sintering Shrinkage and Porosity in Stainless Steel Metal Injection Molding Using Multi-Physics Simulation

**DOI:** 10.3390/ma17235691

**Published:** 2024-11-21

**Authors:** Ying Wu, Kaibo Guo, Junfang Ni

**Affiliations:** 1 Institute of Intelligent Manufacturing and Smart Transportation, Suzhou City University, Suzhou 215104, China; 2 School of Mechanical and Electrical Engineering, Soochow University, Suzhou 215137, China; guokb@suda.edu.cn

**Keywords:** metal injection molding (MIM), sintering shrinkage, particle size distribution, porosity, ANSYS Workbench

## Abstract

In this study, three stainless steel materials (17-4PH, 316L, and 304) were experimentally simulated using metal injection molding (MIM) technology to explore the size shrinkage behavior and defect formation mechanism of materials with different particle sizes during sintering. The sintering environment was linearly heated to 1250 °C at a rate of 5 °C/min and kept warm for 90 min. Multi-physics field coupling analysis was performed using ANSYS Workbench software. Two different regions were selected to simulate the total deformation trend of the material during sintering. The simulation results were compared with data from SEM and EDS analyses to elucidate the influence of particle size on shrinkage behavior and defect distribution. The findings indicate that the gaps between particles far away from the gate position became larger, the degree of densification decreased, the porosity was higher, and the number of white dot inclusions increased. Among the three materials, 17-4PH, which had the smallest particle size, had a greater sintering driving force, a better degree of densification, a smaller predicted total deformation, and a higher shrinkage rate, which is consistent with the hardness test data and the actual density data. In addition, the densification advantage of small particle size powder is not only related to surface energy but is also closely linked to the uniformity of its microstructure. The analysis in this study further promotes the performance optimization of stainless steel materials, indicates a scientific basis for future process improvements and high-precision parts manufacturing in MIM technology, and points to the development direction for high-performance materials.

## 1. Introduction

Stainless steel has become the material of choice for medical devices, surgical tools, and implants due to its superior corrosion resistance and biocompatibility [1,2]. At the same time, in the field of food processing and storage, stainless steel’s high-temperature resistance and resistance to corrosion ensure food safety and hygienic conditions. However, these applications place strict demands on the forming accuracy of stainless steel parts. However, in the actual forming process, process defects such as dimensional inaccuracies and porosity are often encountered, which significantly affect the performance of the material in complex use environments. For example, Zheng et al. [3] prepared 316L stainless steel by laser powder bed fusion (LPBF) technology and found that changes in energy density have an important influence on material density and porosity defects, which are difficult to be effectively controlled in traditional processes. Similarly, Sahoo and Goswami [4] systematically analyzed the causes of porosity defects in the casting process and pointed out that the precise control of temperature and fluidity play a key role in reducing porosity, but traditional casting has obvious limitations in this regard. With the increasing demand for precision and complex-shaped parts, metal injection molding (MIM) has become a necessary method for stainless steel processing. MIM technology combines the flexibility of injection molding with the material advantages of powder metallurgy to produce high-quality products. Precision, high-complexity, and high-performance parts meet strict tolerance requirements, and they can also significantly improve production efficiency and reduce costs [5,6].

MIM mainly includes four stages: mixing, injection molding, debinding, and sintering [7,8]. Various problems can occur in these processing stages. For example, in the injection molding stage, uneven injection speed or unreasonable mold design may lead to incomplete filling and bubble defects [9,10,11]. The problems in the debinding stage are mainly adhesive residue and component deformation. When the temperature or debinding time is not properly controlled, the binder content will be high, and more pores will be generated during the debinding process thus affecting the subsequent sintering stage [6,12,13]. Before sintering, if the green part is not properly handled, it can cause severe shrinkage or deformation during the sintering process. When the temperature and sintering time are not accurately controlled, the porosity will increase, and the strength will decrease [14,15,16]. Therefore, this study simulated the causes of dimensional shrinkage and defects and studied the control mechanism of molding quality, which is the key to ensuring the consistency of product quality and design development [17,18,19], as well as being crucial to the precision and mechanical properties of the final product.

In the past, the methods for predicting the quality of MIM-finished products mainly included experimental testing and simulation [18,20,21,22,23]. For example, Berladir et al. used ANSYS Workbench to simulate the behavior of composite materials during pressing and subsequent sintering, predicted the shrinkage and deformation of the materials, and optimized the process parameters to improve the accuracy of the parts [24]. Trad et al. used finite element analysis methods to predict and evaluate the degree of powder segregation after injection through numerical analysis, simulation, and verification. However, these studies paid more attention to the accuracy of simulation results [25] and less attention to the specific causes of these phenomena. This tendency is more common in the research of the MIM process. Most studies focus on using simulation results to optimize process parameters but lack an in depth exploration of the microscopic mechanisms that cause shrinkage, such as the particle size distribution of the material, internal stress distribution, etc.

In this study, MIM technology was used to process samples of three stainless steel materials: 17-4PH, 316L, and 304. ANSYS Workbench was used to set the molding process parameters and conduct multi-physics field coupling analysis to simulate the dimensional shrinkage of these three materials during the MIM sintering molding stage. With the help of high-precision characterization methods, such as scanning electron microscopy, energy-dispersive spectroscopy, and Vickers hardness testing, the flow behavior, filling mode, and defect distribution of the materials, as well as the formation mechanism of microscopic defects during sintering, were analyzed in depth. Through multi-physics simulation and experimental verification, the research system revealed the influence of particle size on the densification and dimensional stability of stainless steel powder during MIM sintering. The research results provide a scientific basis for optimizing the particle size parameters in material formulation design, help improve the dimensional accuracy and density of materials, and lay the foundation for the development of high-performance MIM materials in the future. This achievement not only promotes the performance optimization of stainless steel materials, but also provides a reference for defect control and mechanical property improvement of other metal materials in the MIM process and expands the application prospects of materials science in complex structures and micro-defect control.

## 2. Materials and Methods

### 2.1. Powder and Feedstock

**Figure 1:**
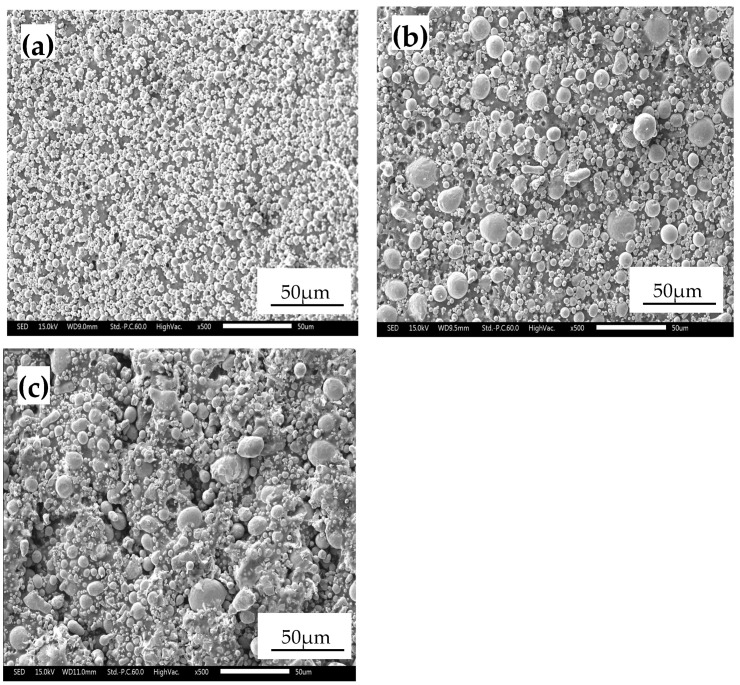
SEM images of the (**a**) 17-4PH powder, (**b**) 316L powder, and (**c**) 304 powder.

In this experiment, three stainless steel powders produced by Tianyuan Powder Co., Ltd. (Zhenjiang, China) were used, with a powder-to-binder ratio of 92:8 (mass%): 17-4PH, 316L, and 304. Their morphology is shown in Figure 1. The particle size distribution of each powder was measured using the Malvern Mastersizer 3000 laser particle size analyzer (Malvern Panalytical, Malvern, UK; website: https://www.malvernpanalytical.com, accessed on 20 June 2024). As shown in Table 1, the 304 powder had the largest particle size and the largest average particle size, with relatively large particles and a wide distribution range; the 17-4PH powder had the smallest particle size, with relatively small and uniform particles overall; and the 316L powder had a medium particle size, with the particle size distribution concentrated in the medium range. The particle size analysis results of the three materials helped in understanding the distribution of the powders and had a certain impact on the quality of the part molding during the injection molding and sintering stages [26].

### 2.2. Metal Injection Molding (MIM)

To systematically compare the formation defect generation mechanisms of these three materials, we strictly regulated the sintering process parameters in this study to exclude the interference of other process variables on the results and ensure the repeatability of the results. Specifically, the sintering process adopts a segmented heating method to control thermal stress and optimize the densification behavior of the material [7,14,27]. The heating stage is set to linearly increase the temperature to 1250 °C at a rate of 5 °C/min and keep it at this temperature for 90 min to ensure that the material is fully densified and structurally stable during the sintering stage. After the insulation, the temperature is slowly cooled at the same rate (5 °C/min) to minimize the internal stress during the cooling process. The strict control of these parameters played a key role in the study, ensuring the reliability and repeatability of the experimental results, and providing a scientific basis for the performance comparison of materials with different particle sizes in subsequent studies.

### 2.3. FEA Simulation

**Figure 2:**
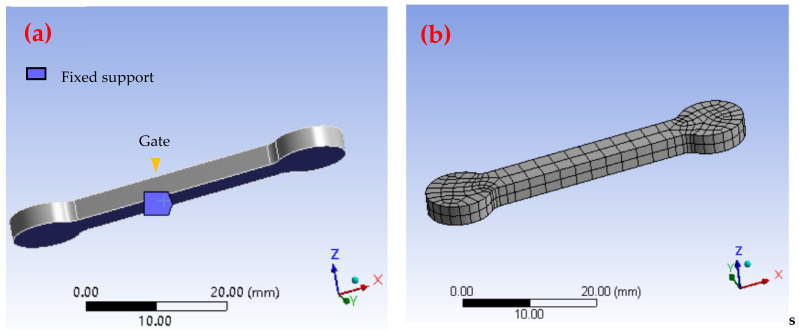
The fixed support surface: gate (**a**) and ANSYS model (**b**).

Finite element analysis (FEA) is a numerical analysis method that approximates various physical field problems by decomposing complex continuous media into a finite number of elements. The sintering process in MIM exhibits typical multi-physics coupling behavior involving multiple physical phenomena, such as heat conduction, material flow, chemical reaction, phase change, and stress–strain interaction. ANSYS Workbench is used for coupling analysis to simulate the influence of the temperature distribution on the diffusion rate, phase change behavior, and volume shrinkage of the material, as well as the generation of internal stress, and predicting the formation of deformations and defects [22,28,29]. As the gate position was in the upper middle position of the part and a reasonable heat deformation state was simulated during the simulation process, the fixed constraint surface was set on the lower surface of the part, as shown in Figure 2.

At the same time, the material properties were set as shown in Table 2 in order to accurately predict the behavior of the three stainless steel materials during the MIM sintering process, and the experimental results were then compared to ensure that the simulation results remained accurate at different temperatures and in different mechanical environments.

### 2.4. Size Variation Mechanisms in MIM

In the MIM manufacturing process, the molding accuracy of parts is affected by many factors. The sintering stage is the stage with the greatest impact on the molding shrinkage and dimensional change, and it is also a key step in part molding. If the molding size and shape accuracy are not properly controlled, it will have a direct impact on its mechanical properties. The dimensional shrinkage that occurs during the sintering process is usually expressed as follows:

(1) $$S=\frac{L_{mold}-L_{final}}{L_{mold}}\times 100\%,$$

where S is the dimensional shrinkage rate, $L_{mold}$ is the mold size, and $L_{final}$ is the final size after sintering. According to the classical sintering theory, during the solid-state sintering process, the material between the particles is redistributed through the diffusion mechanism, resulting in a reduction in the interparticle gaps and the shrinkage of the overall material [15,17,30]. Therefore, the relationship between the dimensional shrinkage rate and the particle size d of the powder and the sintering temperature and time t can be expressed as follows:

(2) $$\Delta L=\frac{k\cdot \gamma \cdot t^{\frac{1}{3}}}{d}.$$

where ΔL is the dimensional change, k is the material constant, γ is the surface energy of the particles, t is the sintering time, and d is the average particle size of the metal powder. It can be concluded from the formula that a smaller particle size means a larger surface area and a higher sintering driving force, which leads to larger dimensional shrinkage, and the dimensional shrinkage gradually increases with an increase in sintering time [16,19,31]. In order to accurately evaluate the influence of different particle sizes on the dimensional shrinkage rate and molding size, the sintering temperature, time, atmosphere, pressure, and other process conditions were unified. Under this condition, the dimensional shrinkage rate can be re-expressed as follows:

(3) $$S\approx \frac{k^{\prime }\cdot \gamma \cdot t^{\frac{1}{3}}}{L_{mold}\cdot d},$$

where $k^{\prime }$ is a comprehensive coefficient that includes the influence of material constants and process conditions (i.e., a constant under uniform conditions) and ignores the differences in material properties. Combining Formulas (2) and (3) reveals that powders with smaller particle sizes tend to produce larger internal stresses during sintering due to their higher specific surface areas and stronger sintering driving forces. This stronger driving force tends to cause larger internal stresses during sintering, which will lead to larger dimensional deformation [30,32,33]. This phenomenon was common in the three stainless steel materials, 17-4PH, 316L, and 304, indicating that particle size plays a dominant role in sintering deformation behavior, and the effect was found to be consistent in the different materials.

### 2.5. Characterization Methods

When studying the sintering behavior of the three stainless steel materials, ANSYS Workbench was first used for a simulation analysis to predict the behavior of the materials with different particle sizes during the sintering process and identify any possible defects. A three-axis coordinate measuring machine (CMM) (Hexagon Leitz Reference) was then used to accurately measure the geometric dimensions of the samples in the XYZ directions for verification, and the dimensional shrinkage rate S was obtained using Formula (1). It should be noted that the pores inside the material will gradually close under the condition of high temperature during the sintering process, resulting in a change in the volume shrinkage rate of the material. Therefore, it can be seen that the porosity is closely related to the shrinkage rate [23,30]. The porosity can be obtained using the following formula:

(4) $$P_{x}=1-\frac{\rho_{Archimedes}}{\rho_{theoretical}},$$

where $P_{x}$ is the porosity, $\rho_{Archimedes}$ is the density measured by the Archimedean method, and $\rho_{theoretical}$ is the theoretical density. The density of the sample was measured by using the Archimedean method in accordance with the ASTM B962-17 standard [34], the morphology and distribution of the pores inside the sample were observed using a Zeiss Sigma 300 scanning electron microscope (SEM), and the hardnesses of the three different materials was tested according to the ASTM E92-17 test standard [17,35,36] to comprehensively evaluate the microscopic mechanism of the material shrinkage.

## 3. Results and Discussion

### 3.1. FEA Results

During the metal injection molding (MIM) process, the metal powder and binder are mixed in a certain proportion to form a material with good fluidity. The material is molten after being heated by the injection machine and injected into the pre-designed mold cavity through the gate under high pressure [11,25]. The gate position is the starting point of the material flow. During injection, this area will be filled first, and the densification process will then begin. Because the material flow path is short, the flow resistance is small, and the temperature is high, the particles are closer and the porosity is lower. Due to the action of pressure, the material flows through the complex mold cavity to each part. With the increase in the flow channel length and the action of complex geometric shapes, such as corners and bifurcations, the flow resistance also increases. Moreover, the fluidity and shear force of the material are significantly weakened. In addition, in the process of filling the mold, differences arise between different areas of the mold: the area close to the gate position has a higher temperature, the material fluidity is better, and the viscosity is reduced, whereas in the area far away from the gate position, the temperature is lower, the material fluidity decreases, and the viscosity increases [13,28,37]. This temperature gradient will also lead to an uneven distribution of material fluidity and shear force. Therefore, under the influence of comprehensive factors, the gaps between particles are slightly larger, the tightness is insufficient, and the porosity is high, especially in the area far away from the gate, which makes it impossible for this area to fully fill the mold like the material near the gate.

**Figure 3:**
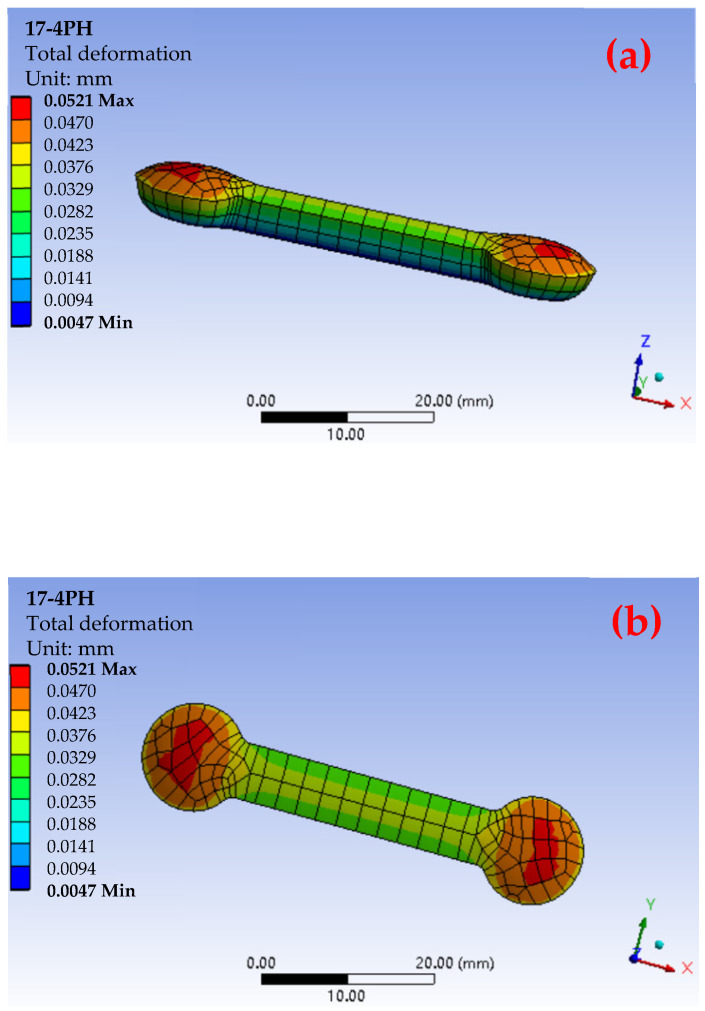
A magnified view of the 17-4PH results: total deformation side (**a**) and gate side (**b**).

ANSYS Workbench was used to simulate the total deformation distribution cloud of the 17-4PH material after the MIM process and sintering, as shown in Figure 3. Because the gate position is located in the middle of the upper surface of the part and the lower surface is a fixed surface, after heating, the upper surface expands without enough downward release space and will be released through upward deformation, so the thermal expansion effect is mainly concentrated on the upper surface, resulting in bulging and bending deformation of the upper surface [16,31,32]. The area near the middle gate position is more fully filled due to the large shear force and good fluidity during the injection molding process. Because the material is well densified, there are fewer internal pores and stronger internal support of the material, so the deformation was small during the sintering process. However, in the area far from the gate, due to the weakened material fluidity and insufficient shear force, the filling is incomplete, the porosity is high, and it is more likely to produce uneven shrinkage at high temperature. The stress concentration phenomenon is obvious, resulting in uneven shrinkage. The maximum total deformation reaches 0.0521 mm [38,39].

**Figure 4:**
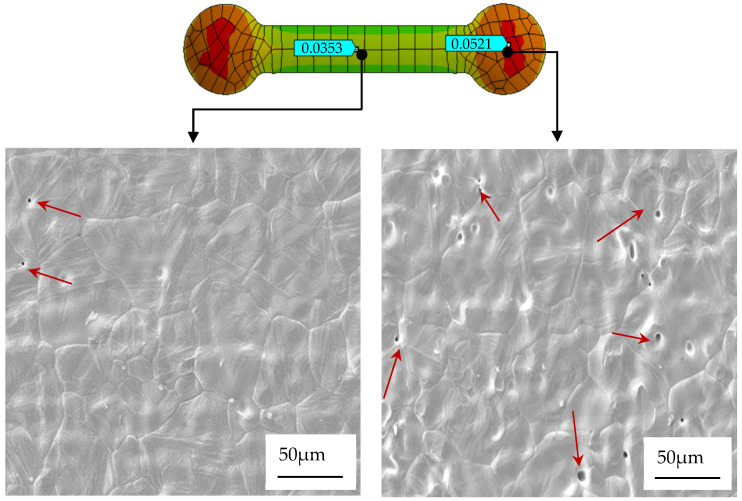
SEM images of 17-4PH’s near gate and far gate areas. The arrows point to the pores observed in the material.

In order to further verify the above simulation results, the microstructural characteristics after sintering were compared through scanning electron microscopy (SEM). As shown in Figure 4, in the area close to the gate, the microscopic porosity of the material is significantly lower, the density between particles is higher, and the surface is flatter and smoother, which is consistent with the simulation prediction; that is, the total deformation in this area is small, and the maximum total deformation is about 0.0353 mm. In the area far away from the gate, due to incomplete filling and low density, more pores appear in the SEM image, confirming the cause of the large deformation observed in this area during the simulation.

Through multi-physics field coupling analysis, the densification and deformation laws of different regions during the sintering process were successfully captured in the simulation, which is highly consistent with the actual SEM observation results. This approach not only accurately predicts the macroscopic deformation, but also reflects the changes in particle structure and pore characteristics at the micro level.

### 3.2. Sintering Behavior and Particle Size Effects

In this study, it could be seen from the analysis that 17-4PH had a small and uniform particle size, 316L had a relatively medium particle size, and 304 had a slightly larger particle size and uneven distribution, as shown in Table 1. During the sintering process, materials with smaller particle sizes will produce stronger sintering driving forces under high-temperature conditions due to their larger surface areas, so the interfaces between particles can be shortened and fused more effectively. This effect enables small-particle materials to quickly reduce pores during the sintering process and form a denser structure, where the porosity is small and the air can be discharged in time, as shown in the SEM image of Figure 4. As sintering progresses, the temperature gradually increases. Due to the reduction in porosity, the stress distribution of the 17-4PH material is more uniform, and the internal thermal stress concentration phenomenon is effectively alleviated, so the total deformation after sintering is reduced [32,40].

**Figure 5:**
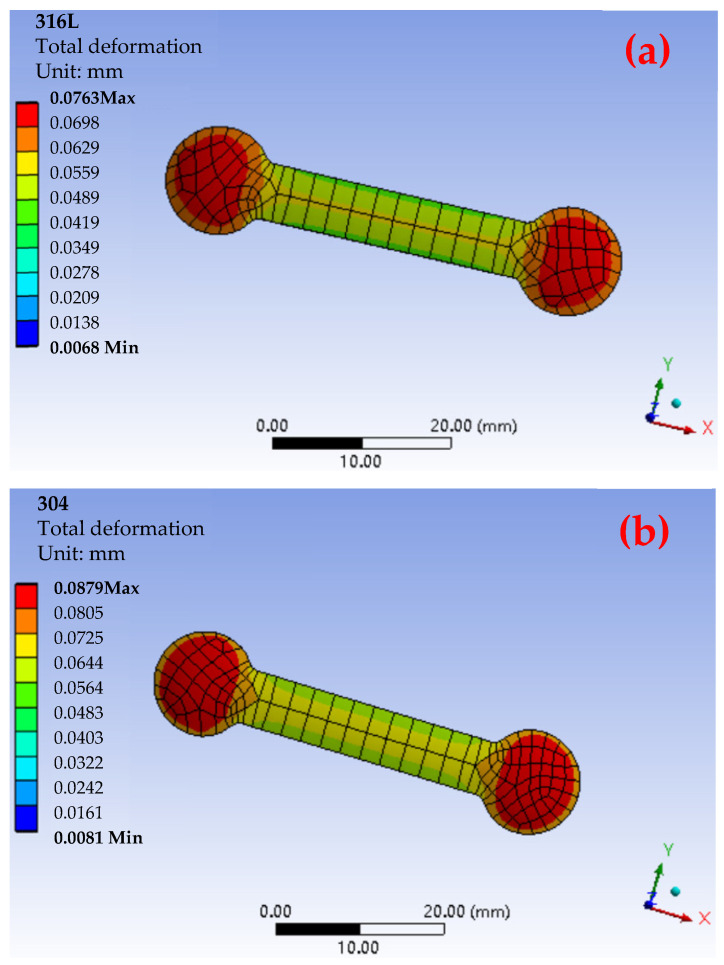
Magnified results of the total deformation of 306L (**a**) and 304 (**b**).

**Figure 6:**
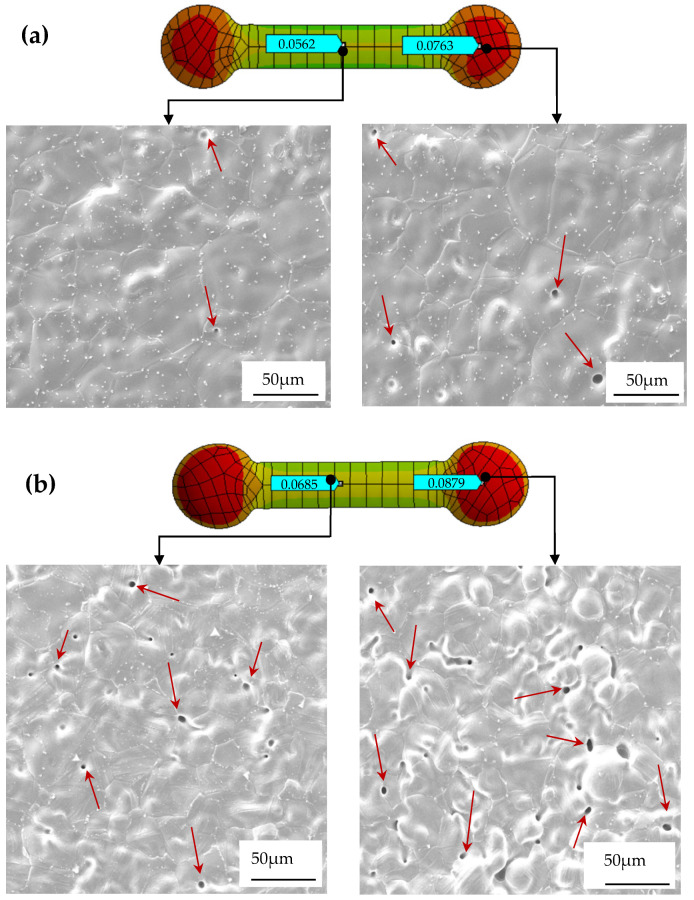
SEM images of 316L (**a**) and 304 (**b**) materials’ near and far gates. The arrows indicate the pores observed in the material.

To evaluate the difference in total deformation performance of materials with different particle sizes, as shown in Figure 4 and Figure 5, the ANSYS simulation results show that there were fewer red high-strain areas in the total deformation cloud map of the small-particle materials, indicating that the internal stress of the material had been effectively dispersed. In contrast, its surface area gradually decreased with the increase in particle size, resulting in an insufficient sintering driving force, insufficient bonding between particles during sintering, and high porosity. Among the three materials, the maximum total deformation of the 316L material reached 0.0763 mm, while the maximum total deformation of 304 material was 0.0879 mm. In contrast, the total deformation of the 17-4PH material was much smaller, with a maximum value of 0.0521 mm. These values showed that the large-particle 304 material underwent greater deformation after sintering, while the small-particle 17-4PH material had the smallest deformation. To further verify the validity of the simulation results and the characteristics of large-particle materials with higher porosity due to insufficient bonding between particles during sintering [17,31], Figure 6 shows the simulation results of the total deformation of the 316L and 304 materials near and far from the gate area and the corresponding scanning electron microscope (SEM) images. It can be observed that the number of pores in the material near the gate area was significantly higher than that in the material far from the gate area, especially in the area far from the gate. The pores marked by red arrows in the figure show that the 304 material had a higher porosity after sintering, while the number and size of pores in the 316L material were relatively small, but still significantly higher than those in the 17-4PH material. 

**Figure 7:**
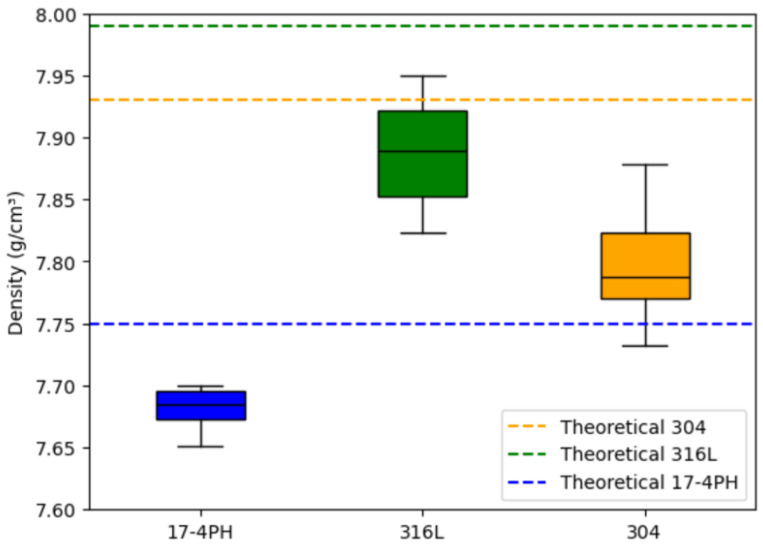
Comparison of actual and theoretical densities of 17-4PH, 316L, and 304.

The actual density of the three materials was measured by the Archimedean method [19,41] and analyzed in conjunction with the data in Figure 7. The results show that the actual density range of the 17-4PH material is 7.65–7.70 g/cm^3^, which is slightly lower than its theoretical density of 7.75 g/cm^3^. According to Formula (4), the porosity range of 17-4PH is calculated to be 0.65–1.29%, with a small dispersion in density values, indicating low and evenly distributed porosity. In comparison, the actual density range of the 304 material is 7.74–7.89 g/cm^3^, which is significantly lower than the theoretical density 7.93 g/cm^3^. The porosity range is 0.50–2.40%. The density dispersion is large, reflecting higher and uneven pore distribution. The actual density range of the 316L material is 7.82–7.95 g/cm^3^, which is close to its theoretical density of 7.99 g/cm^3^. The porosity range is between 0.50 and 2.13%. The density value dispersion is moderate, showing moderate density and pore distribution. Experimental results show that there are still certain pores inside the sintered material. These unresolved pores not only reduce the density of the material, but also hinder the discharge of gas, resulting in a relatively low dimensional shrinkage rate [18,30]. At the same time, due to the existence of pores and stress concentration areas, the material produces varying degrees of total deformation after sintering. The simulation results in Figure 3 and Figure 5 further verify this: the maximum total deformation of the 17-4PH material is 0.0521 mm, displaying a smaller red high-strain area, consistent with its lower porosity. In contrast, the 304 material exhibits a maximum total deformation of 0.0879 mm, with a prominent red high-strain area, indicating that larger porosity brings greater deformation; the total deformation of the 316L material is between the two, with the maximum deformation of 0.0763 mm, and the high-strain area shown in the simulation diagram is also relatively moderate.

### 3.3. Shrinkage Behavior Analysis

High temperatures during the MIM sintering process promote the diffusion of particles and grain boundary migration, achieving densification. As the sintering temperature increases, diffusion occurs inside the material, the particles gradually combine, the pores gradually close, and a dense material is finally formed. However, white, small, round, or irregular bright spots are often produced during the sintering process due to incomplete pore filling, oxide formation, or impurity enrichment. These white spots will hinder the densification of the particles, resulting in an increase in internal porosity, which, in turn, affects the dimensional shrinkage of the sintered product [33,42,43].

**Figure 8:**
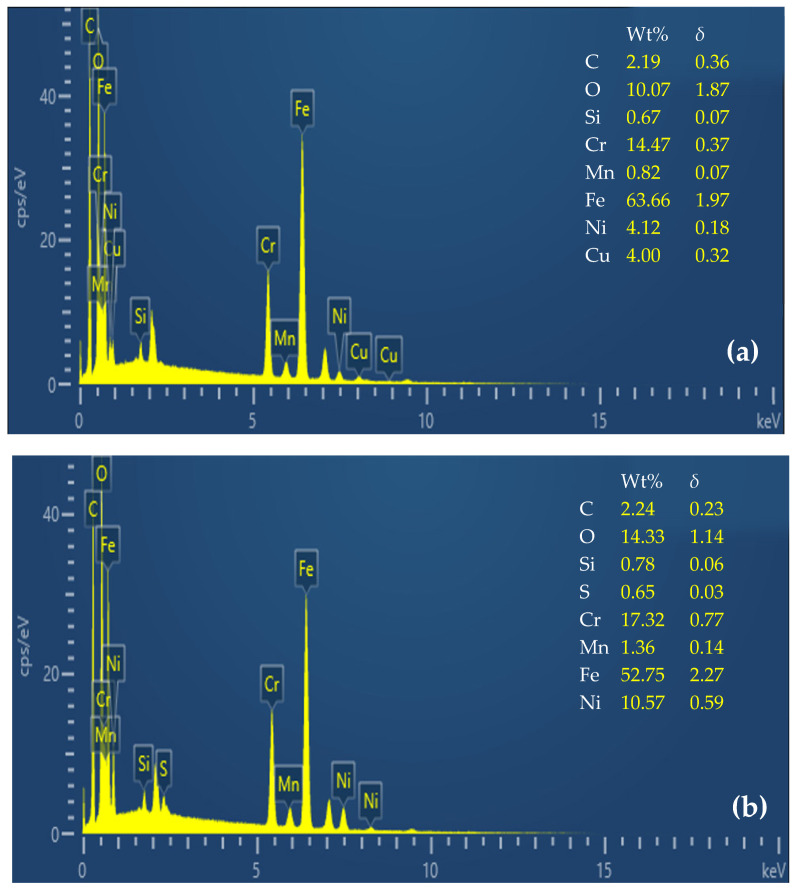

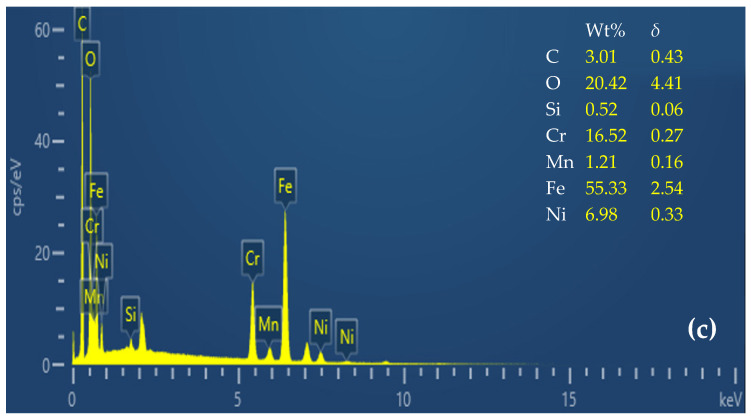
EDS spectrum analysis of 17-4PH (**a**), 316L (**b**), and 304 (**c**).

As shown in Figure 8, for 17-4PH, with a small particle size and uniform distribution, the white dot-like material increased at a position far from the gate as the material fluidity decreased and the degree of sintering densification decreased. The EDS spectrum analysis showed that the mass percentage (Wt%) of iron (Fe) was lower than the normal range, and the standard deviation (*δ*) corresponding to Wt% was 1.97. This indicates that, after sintering, the distribution of iron was more uneven than that of other elements [10,44]. In comparison, the mass percentage of iron in the medium-sized 316L and large-sized 304 also decreased after sintering, and the *δ* increased to 2.27 and 2.54, respectively, indicating that the distribution of iron in these areas fluctuated more. With the increase in white dot-like material at both ends of the sample, the porosity also increased accordingly, which hindered the effective diffusion of the particles and further aggravated the insufficient filling of pores inside the material.

**Figure 9:**
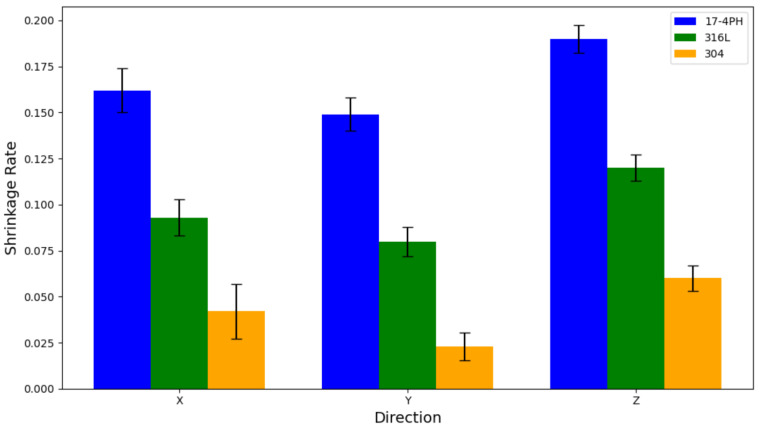
Dimensional shrinkage and standard deviation of 17-4PH, 316L, and 304 stainless steel in the X, Y, and Z directions.

In order to further study the relationship between porosity and dimensional shrinkage, the sintered 17-4PH, 316L, and 304 stainless steel samples were measured in the XYZ directions by a three-dimensional coordinate measuring machine (CMM), as shown in Figure 9. The measurement accuracy was 0.001 mm. Multi-point measurements were taken at key positions of each sample in the XYZ directions, such as the end points and midpoints, and 20 data points were collected to ensure the accuracy and repeatability of the measurement. The dimensional shrinkage of the three materials was calculated according to Formula (1). The results indicate that the 17-4PH material has the highest dimensional shrinkage due to its lower porosity and higher density, especially in the Z direction, where the average shrinkage reached 0.182; while the dimensional shrinkage of the 304 material was the smallest, with a shrinkage of only 0.058 in the Z direction; and the shrinkage of the 316L sample was between the two, with a shrinkage of about 0.122 in the Z direction. Analysis of the data in the figure shows that, due to the greater compression in the Z direction during the mold pressing process, the initial density between the particles is higher, and the voids are eliminated more during the sintering process. Therefore, the degree of densification in the Z direction is the highest and the shrinkage rate is relatively the largest. In contrast, the X and Y directions are more susceptible to obstruction during injection molding and sintering due to the longer material flow paths, resulting in increased porosity, making the shrinkage rate in the X direction slightly lower than that in the Z direction. For example, the X direction shrinkage rate of 17-4PH is approximately 0.161. In addition, due to the longer flow path in the X direction, the resistance generated during injection and sintering is greater, resulting in an increase in porosity, which in turn increases the standard deviation of the X direction measurement data. In contrast, the flow path in the Z direction is relatively short, and the material is easier to flow and densify, so the standard deviation is smaller [11,45,46]. On the other hand, the presence of these white dot-like substances exacerbated the unevenness of the surface. For the 304 material, with an uneven particle size distribution, large and small particles densified at different rates during the sintering process, resulting in uneven sintering and incomplete pore filling. This caused the sample surface to show obvious uneven brightness, especially in some local areas where the brightness was higher, indicating that these areas had significant protrusions or pores, resulting in an uneven surface and the formation of more white dot-like substances [47].

The abnormal enrichment of elements hindered the effective diffusion between particles, affected the bonding of particles, and led to higher porosity and the generation of a large number of white spots, which aggravated the surface unevenness and further reduced the densification level of the material. It was found that with the increase in particle size, the diffusion path of the large-size particles became longer during the sintering process, and the pores between particles were not easy to close, resulting in more pores remaining, which caused the increased generation of white spots. In addition, the size shrinkage rate showed a downward trend.

### 3.4. Mechanical Properties

**Figure 10:**
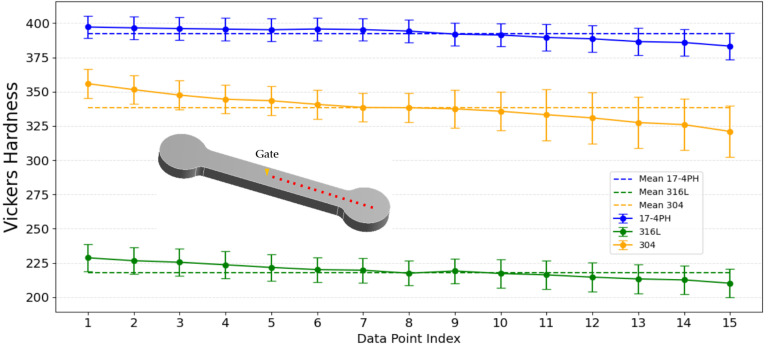
The Vickers hardness analysis results and the standard deviation from the near gate to the far gate.

According to the simulation results of ANSYS Workbench, the deformation was large at the position far from the gate. Therefore, the hardness of the three samples was measured along the straight line from the gate to the far end of one side. Fifteen equidistant measurement points were selected, and the Vickers hardness values were recorded at each point. It was found that the overall hardness of the 17-4PH material was higher and the hardness difference between near the gate and far from the gate was small, as shown in Figure 10. The hardness data ranged from 383.25 HV to 397.21 HV, with an average of 392.28 HV and a standard deviation of 4.36. The hardness distribution was relatively uniform, and the sintering density was good. SEM observation of the 304 material showed that there were more pores or non-dense areas on the surface. From the experimental data, it could be seen that the 304 material had a higher overall hardness than the 316L material, and the hardness varied greatly, ranging from 321.02 HV to 355.87 HV. The hardness of the area far from the gate decreased significantly, with an average of 338.15 HV and a standard deviation of 9.29, indicating that the hardness distribution was uneven and the hardness of the area far from the gate decreased significantly. The hardness of the 316L material was relatively low, with an average hardness of 219.16 HV. The hardness data ranged from 210.21 HV to 228.78 HV. There was also an obvious trend, where the hardness near the gate was higher than the hardness at both ends. The standard deviation of 5.22 showed that, compared with the 304 material, the hardness distribution value was more concentrated [14,40,48].

It was found that the 17-4PH material showed the most stable and uniform hardness distribution, indicating that its sintering effect was the best. The 316L material had a more uniform hardness distribution, smaller changes, and better sintering quality. The hardness of the 304 material changed significantly, especially at locations far away from the gate, which proves that the degree of densification during the sintering process was poor and that there was more porosity and uneven hardness.

### 3.5. Comparative Analysis of Shrinkage, Porosity, and Mechanical Properties

To reveal the relationship between material properties and the MIM process performance, as well as to provide a scientific basis for material selection, the above studies have been consolidated, systematically comparing the shrinkage, average porosity, and Vickers hardness of three stainless steel materials (17-4PH, 316L, and 304) processed by MIM in different directions. The differences in these properties between the materials are analyzed, highlighting the influence of particle size, sintering conditions, and material composition on the final properties of MIM parts.

It can be clearly seen from Table 3 that the 17-4PH material has a finer and more uniform particle size, and has undergone significant densification during the sintering process, forming a low porosity characteristic, and after sintering, it shows the highest shrinkage in all three directions. At the same time, due to the high degree of densification, it has a higher shrinkage and a lower average porosity. This uniform shrinkage and low porosity enhance the mechanical properties, making the hardness distribution of the entire sample more consistent. The 316L material has moderate shrinkage and porosity, showing a moderate hardness distribution and a relatively stable mechanical profile. However, the 304 material has a relatively low degree of densification due to its large and uneven particle size. Among the three materials, it shows the lowest shrinkage and the highest porosity, reflecting the lower and less uniform internal structure density. Although the hardness value of 304 is higher than that of 316L, its hardness value fluctuation range is the largest among the three materials.

## 4. Conclusions

This study comprehensively analyzed the dimensional shrinkage behavior of three stainless steel materials during the MIM sintering process. The three stainless steel samples manufactured by MIM were characterized and analyzed through ANSYS Workbench. The analysis showed that the material particle size has a significant impact on the sintering densification and porosity distribution. Materials with smaller particle sizes (such as 17-4PH) show a higher sintering driving force and better density due to their larger surface area, and their size shrinkage is more uniform. In comparison, materials with larger particle sizes (such as 316L and 304) have an insufficient sintering driving force and higher porosity, resulting in a poor densification effect and uneven size shrinkage. When combined with the hardness data, the mechanical properties were also found to be relatively poor. Additionally, the deformation results of these three materials simulated by ANSYS Workbench showed that the farther away from the gate, the greater the deformation. Scanning electron microscopy, energy-dispersive spectroscopy, and a three-axis coordinate measuring machine were used to verify the areas with higher porosity and white dots. The surface of the sample became uneven due to increased inclusions and uneven particle size distribution. These findings provide an important reference for optimizing the particle size and formulation design of stainless steel MIM materials, particularly in controlling the densification and defect formation mechanism during the sintering process under different particle size conditions. Future research can further deepen the following aspects: 

(1) Examining the effects of different particle sizes and particle distributions on the microstructure and mechanical properties of stainless steel materials and providing more ideal material design parameters for the MIM process.

(2) Studying the interaction of multi-component stainless steel powders during the sintering process to improve the densification effect and durability of the material.

(3) Developing new high-performance stainless steel alloys and optimizing materials for the MIM process to meet the manufacturing needs of complex shapes and high-precision parts. In addition, the sintering process conditions can be appropriately optimized, such as optimizing the temperature curve and pressure field, to further improve the material performance. These research directions will lay the foundation for the application of MIM technology in high-performance stainless steel materials.

## Figures and Tables

**Table 1:** A summary of the particle size data for the 17-4PH powder, 316L powder, and 304 powder.

Powder	D10 (µm)	D50 (µm)	D90 (µm)	Mean Particle Size
17-4PH	2.47	6.03	10.12	6.87
316L	3.12	7.54	12.08	8.91
304	4.11	8.48	14.37	9.65

**Table 2:** Material properties.

Property	17-4PH	316L	316L
Density (g/cm^3^)	7.75	7.99	7.93
Elastic Modulus (GPa)	200	193	193
Poisson’s Ratio	0.27	0.30	0.29
Thermal Expansion Coefficient (×10^−6^/°C)	10.8	16.5	17.2
Specific Heat Capacity (J/kg·K)	460	500	500
Thermal Conductivity (W/m·K)	15.0	16.3	16.2

**Table 3:** Dimensional shrinkage, porosity, and hardness of 17-4PH, 316L, and 304 stainless steels.

Property	17-4PH	316L	316L
Dimensional Shrinkage (X)	0.161	0.087	0.044
Dimensional Shrinkage (Y)	0.149	0.081	0.024
Dimensional Shrinkage (Z)	0.182	0.122	0.058
Porosity (%)	0.65–1.29	0.50–2.13	0.50–2.40
Average Vickers Hardness (HV)	392.28	219.16	338.15
Hardness Standard Deviation (HV)	4.36	5.22	9.29

## Data Availability

The original contributions presented in the study are included in the article, further inquiries can be directed to the corresponding authors.
